# Auditory Processing in Stuttering Children: Behavioral and Electrophysiological Assessments

**DOI:** 10.1055/s-0045-1804518

**Published:** 2025-07-01

**Authors:** Letícia Gregory, Milaine Dominici Sanfins, Piotr Henryk Skarzynski, Rafael Fabiano Machado Rosa, Paulo Ricardo Gazzola Zen, Pricila Sleifer

**Affiliations:** 1 Postgraduate Program in Pathology, Universidade Federal de Ciências da Saúde de Porto Alegre, Porto Alegre, RS, Brazil; 2 Discipline of Hearing Disorders, Department of Speech Therapy, Escola Paulista de Medicina, Universidade Federal de São Paulo, São Paulo, SP, Brazil; 3 Department of Teleaudiology and Screening, Światowe Centrum Słuchu Instytutu Fizjologii i Patologii Słuchu, Nadarzyn, Poland; 4 Instytut Narządów Zmysłów, Kajetany, Poland; 5 Department of Pathology, Universidade Federal de Ciências da Saúde de Porto Alegre, Porto Alegre, RS, Brazil; 6 Department of Internal Medicine, Universidade Federal de Ciências da Saúde de Porto Alegre, Porto Alegre, RS, Brazil; 7 Department of Health and Human Communication, Instituto de Psicologia, Universidade Federal do Rio Grande do Sul, Porto Alegre, RS, Brazil

**Keywords:** stuttering, event-related potentials, P300, hearing, children, auditory evoked potentials

## Abstract

**Introduction** Stuttering is a human communication disorder characterized by involuntary interruptions to speech flow. Electrophysiological tests and behavioral evaluations allow the neuroaudiological processes involved in stuttering to be investigated.

**Objective** To investigate group differences in the central auditory system using long-latency auditory evoked potentials, cognitive potentials, and behavioral assessments in children who stutter and compare them with fluent children.

**Methods** We assessed 18 children with stuttering and compared them with a control group of 18 children without speech or language impairment. All children were evaluated with pure tone and speech audiometry, acoustic immittance measures, brainstem auditory evoked potentials, long latency auditory evoked potentials, cognitive potentials, and behavioral tests of auditory processing – Random Gap Detection Test (RGDT), Dichotic Digit Test (DDT); Duration Pattern Test (DPT); Pediatric Speech Intelligibility (PSI); and Masking Level Difference (MLD). The Scale of Auditory Behaviors (SAB) questionnaire was also applied.

**Results** Children who stuttered had longer latencies of the P2 and P3 waves compared with the fluent group. There was no difference in P2 amplitudes, but there was a smaller P3 amplitude in children who stuttered, and they also showed significant alterations in the DDT and DPT. Furthermore, an association was found between increased P2 and P3 latency and SAB scores.

**Conclusion** The present study indicates that children who stutter tend to have decreased auditory ability in terms of central auditory processing, and this shows up psychophysically and on the SAB questionnaire.

## Introduction

Stuttering is a disorder characterized by involuntary interruptions that make it difficult to speak fluidly. There are repetitions, prolongations, and blocks, generally occupying more than 3% of speech. 1 2 3 It is common for it to begin in childhood, even before 18 months of age. Around 95% of children develop the disorder before the age of 4, with the peak age being around 33 months. 4

The prevalence of stuttering is estimated to range from 2.2 to 5.6% of the general population, 4 while in preschool-aged children the incidence ranges from 5 to 11%. 5 6 As with other language and speech disorders, males are most affected. In children under five years old, the proportion of boys who stutter is twice as high as that of girls. The difference between the sexes becomes even more pronounced with age, with an increasing proportion of males. 4 7

The cause of stuttering is multifactorial and is linked to neurophysiological and genetic factors. Studies have shown anatomical variations, morphological differences, hemispheric asymmetry, and difficulties in motor, linguistic, and auditory function. 8 9 10 11 12 13 14 15

Speech perception and production are related, and the production of intelligible speech depends on the ability to follow the paradigms of the acoustic spectrum and understand the prosody of the interlocutor's speech. 16 17 Thus, research in this area involves investigating the relationship between auditory function and language disorders, including stuttering. Studies focused on stuttering and hearing have found that, comparing fluent and disfluent individuals, there are distinct differences in both speech processing and the processing of auditory information. 18 19 20 21 22 23 Nevertheless, studies that provide hard physiological evidence are scarce.

One way to objectively verify auditory integrity and functionality is through use of Auditory Evoked Potentials (AEPs), which are traces generated by the bioelectrical activity of the auditory pathways following acoustic stimulation. 24 25 26 27 28 Auditory evoked potential is an objective method that provides qualitative and quantitative information about the auditory pathway through measurement of the latency and amplitude of wave peaks. Measurements can, for example, determine the time it takes for an acoustic signal to travel through the auditory pathway and whether a stimulus is adequately received and interpreted by the auditory cortex. 26

Long-latency auditory evoked potentials (LAEPs) refer to a series of electrical changes that occur in the central nervous system hundreds of milliseconds after stimulation. The bioelectrical responses of the thalamus and cortex are detected in the form of waves, with the sequence P1-N1-P2-N2 being recorded. 27 These exogenous potentials reflect auditory sensitivity at each level, providing information about the integrity of the auditory pathway, neural coding, and perception of acoustic stimuli at the central level. 28 29 Some authors have used the P2 potential as a biomarker for auditory training, as a measure of the subject's auditory discrimination. 30 P2 is a prerequisite for the subsequent generation of the P3 wave, because a target stimulus must first be discriminated in order to be identified. 31 Thus, the study of P2 can help us understand the precognitive processes elicited by non-linguistic tones.

P3 is a LAEP that can be used to electrophysiologically assess hearing processing in the brain, as it reflects cortical activities involved in auditory processing. 24 32 33 34 It is an endogenous potential that can also be called a cognitive potential, as in order for an individual to perform a cognitive task, an active response is first required, such as counting stimuli. 33 34 P3 is a positive peak that appears around 300 milliseconds after active detection of a rare stimulus among frequent stimuli. 24 27 It is a potential that reflects central auditory processes such as auditory discrimination, temporal processing, attention, discrimination, recognition, perception, and auditory memory. 24 27 34 35 Its site of generation has yet to be completely determined, but it is known that the hippocampus, auditory cortex, prefrontal cortex, and centroparietal cortex contribute. 24

There has been some research investigating the relationship between stuttering and P3, but no consensus has yet emerged. Whereas some studies 36 37 38 did not find any differences between stutterers and fluent speakers, others suggest there is some sort of change in the P3 wave in individuals who stutter –either an increase in latency 19 36 39 40 41 or a decrease in amplitude. 40 42 The variability of results can be justified by the sample size, the heterogeneity of the samples, and the fact that most studies are performed with adults, which makes it difficult to assess whether the altered P3 results are the cause or consequence of stuttering.

Given this context, studies are necessary to better understand these responses, to contribute to scientific research and, in the future, with their use in clinical practice, with the aim of assisting in diagnosis and the development of therapeutic programs.

The aim of the present study is to record and analyze LAEPs–waves P2 and P3–and to correlate them with the behavioral assessments of children who stutter, comparing them with responses from fluent children. More generally, we also investigate whether there are any associations between the electrophysiological findings and stuttering severity or between electrophysiology and scores on the Scale of Auditory Behaviors questionnaire.

## Methods

### Subjects

This is a cross-sectional, analytical, and quantitative study. The convenience sample consisted of children aged 7 to 11 years old, of both sexes, divided into 2 groups: an experimental group (EG), consisting of children with developmental mild-to-severe stuttering, and a control group (CG) comprised of children without stuttering, matched by sex and age.

### Inclusion and Exclusion Criteria

The EG was made up of children who spoke Brazilian Portuguese who had complaints of stuttering—but had received no prior treatment—and who had no comorbidities. We excluded those who did not know how to count from 1 to 50, had some type of hearing loss, an altered otorhinolaryngological evaluation, or who failed to perform the proposed procedures.

The CG was made up of children who spoke Brazilian Portuguese and had no history of psychological/psychiatric, neurological, motor, or communication disorders. We excluded those with hearing or language impairment, those who did not know how to count from 1 to 50, had some type of hearing loss, an altered otorhinolaryngological evaluation, or failed to perform the proposed procedures.

### Equipment

a) *Anamnesis* . Information comprising age, sex, dominant hand, and education was collected with a description of any linguistic, social, neural, or otological impairment.
b) *Assessment of stuttering* . To assess the stuttering of the EG patients, an anamnesis of the child was performed, which verified family history regarding stuttering, its emergence, self-perception of the degree of disfluency, feelings about one's own speech, impact of stuttering on life, and daily routine. A speech sample was also collected, with video footage, consisting of spontaneous speech lasting ∼ 5 minutes. The speech was transcribed by the Speech Transcription Protocol, 43 and 200 syllables were analyzed by the Speech Fluency Assessment Protocol, 44 which evaluates the type of disfluencies, speech speed, frequency of ruptures, and physical parameters. Afterwards, the Stuttering Severity Instrument (SSI-4) 45 was applied to classify the severity of stuttering. Finally, a diagnosis of persistent developmental stuttering was made by two speech therapists with experience in the area. To test the agreement of the analysis between the judges, the Cohen's Kappa coefficient was used.
c) *Peripheral audiological assessment*
  c.1) Children from both groups underwent inspection of the external auditory canal, tonal and speech audiometry, and acoustic immittance measurements. Inspection of the external auditory canal was performed with a otoscope (Welch Allyn, Inc., Skaneateles Falls, NY, USA) to rule out the presence of earwax or foreign body.
  c.2) Pure tone audiometry was performed in an acoustically treated cabin, using a Harp audiometer (Inventis), with headphones and a bone vibrator. Hearing thresholds for frequencies from 0.25 to 8 kHz were investigated via air, while those from 0.5 to 4 kHz were investigated via bone. In speech audiometry, the Word Recognition Score (WRS) and Speech Recognition Threshold (SRT) were assessed, both relying on the patient repeating the words presented. To perform the WRS, 25 monosyllabic words were presented at an intensity of 40 dB re.
  c.3) Acoustic immittance measurements were performed using an AT235h audiometer (Interacoustics A/S, Middelfart, Denmark). Ipsilateral and contralateral acoustic reflexes were investigated at 0.5, 1, 2, and 4 kHz in both ears.

All children showed good ability to detect pure tones and had adequate sound transmission through the tympanic-ossicular system, with hearing thresholds better than 15 dB HL at frequencies from 0.25 to 8 kHz, type-A tympanometric curves, and ipsilateral and contralateral acoustic reflexes.

d) *Behavioral tests of central auditory processing skill* . These tests were performed in a soundproof booth, using verbal and non-verbal stimuli presented through headphones connected via the Harp audiometer to a notebook containing the behavioral test tracks. The test battery included the: (d.1) Random Gap Detection Test (RGDT); (d.2) Dichotic Digit Test (DDT); (d.3) Duration Pattern Test (DPT); (d.4) Pediatric Speech Intelligibility (PSI); and (d.5) Masking Level Difference (MLD), following the recommendations of Academia Brasileira de Audiologia. 46 The presentation intensity for all tests was calculated based on the mean air-conduction thresholds at 0.5, 1, and 2 kHz. All tests were performed after the children had been previously trained, ensuring they understood the tasks. For each test, the results were classified as normal or altered, following the standard normality values.
  (d.1) In the RGDT, pure tones were presented paired with short intervals of silence, ranging from 0 to 40 milliseconds, in random order, and presented binaurally. Children had to report how many sounds they heard. The interval detection threshold was the smallest interval from which the child began to consistently identify the occurrence of two stimuli.
  (d.2) The DDT was applied in two stages, binaural separation and integration. In the binaural integration stage, the child heard four numbers, two in each ear simultaneously, and they had to repeat all four digits, regardless of the order of presentation. In the binaural separation stage, two digits were presented in each ear, and the child was asked to repeat only the two digits heard in the selected ear, ignoring the digits in the contralateral ear. Each incorrectly identified digit was equivalent to a 1.25% error, and the number of errors × 1.25 was subtracted from 100%.
  (d.3) In the DPT, 3 1-kHz tones of 2 lengths were presented at intervals of 300 milliseconds. The long tone (L) had a duration of 500 milliseconds and the short one (S) of 250 milliseconds. There were six patterns (LLS, SSL, LSL, SLS, LSS, SLL), which were repeated randomly. The child was asked to repeat the sequence, either naming or imitating it.
  (d.4) In the PSI, the child was given a board containing pictures. Initially, the test pictures were presented for recognition only, and then, later, the child was instructed to pay attention and point to the picture corresponding to the sentence they heard, ignoring a competing message (a story). To begin, the test was applied in the presence of a contralateral competing message in the right ear (at speech/noise ratios of 0 dB and -40 dB) and later in the left ear at the same ratios. In the second stage, the test was applied with an ipsilateral competing message in the right ear (at speech/noise ratios of 0, -10, and -15 dB), and then in the left ear at the same ratios.
  (d.5) In the MLD, 33 segments of narrowband noise were presented to one ear, for at least 3 seconds, in the presence or absence of a 0.5-kHz pure tone. Three conditions were considered: narrowband noise and pure tone in phase in both ears, pure tone in inverted phase in one ear, and noise in phase in both ears, and noise without the presence of the 0.5-kHz tone. The children were instructed to report every time they heard the tone. For analysis, the number of times that the child signaled they heard the tone were added up. This number was transformed into dB, according to a table supplied with the test.
e) *Electrophysiological assessment*
  (e.1) Auditory Brainstem Response (ABR). A click stimulus was applied to verify the integrity of the auditory pathway. A click stimulus was used on the right and left ears separately. There were 27.7 clicks per second, of rarefied polarity and at 80 dB HL, to a total of 2,048 clicks, a recording window of 12 milliseconds, bandpass filter of 0.1 to 3 kHz, and digitization rate of 100 K. Participants were instructed to remain relaxed in an armchair, without speaking and with their eyes closed.
  (e.2) Long latency brainstem auditory evoked potential. For this, the Masbe ATC Plus equipment (Contronic LLC, Huntington Beach, CA, USA) with a 3A earphone (Etymoic Research, Inc., Elk Grove Village, IL, USA) insert was used. The child was seated comfortably in a chair, and their skin was cleaned with exfoliant (Nuprep – Weaver and Company, Aurora, CO, USA) and gauze. Electrodes were then placed with conductive paste (Ten20–Weaver and Company) and secured with adhesive tape. The ground electrode was placed on the forehead, the active electrode (Fz) close to the scalp, the M1 electrode on the right mastoid, and the M2 electrode on the left. The earphones were placed in both ears. Impedance values were kept below 5Ω, and the difference between the 3 electrodes did not exceed 2Ω. An electroencephalogram (EEG) scan was performed to capture spontaneous brain electrical activity and check for artifacts that might interfere with the examination. Children were instructed not to tense their limbs and not to cross their legs and arms.

Tone burst stimuli of 1 or 2 kHz were applied at 80 dB HL. The tone bursts had a 20-millisecond plateau and 5 milliseconds rise and fall times. Each ear was tested individually. For recording P3, rare and frequent stimuli were presented. The child was instructed to pay attention and count rare stimuli, which appeared randomly among a series of frequent stimuli. At the end, the child needed to report how many rare stimuli they had heard. The frequent stimulus had a frequency of 1 kHz, and the rare stimulus 2 kHz, both presented randomly by the computer at a speed of 1.1 stimuli per second, in a rare-frequent (oddball) paradigm, with probabilities of appearance of 20% and 80%, respectively. A filter from 0.5 to 20 Hz and window of 900 milliseconds were used. The amplitude and latency values were obtained by looking at the two traces and identifying the highest peak. To identify P3, the largest positive polarity peak after the N1-P2-N2 complex was considered. The P3 component was identified by subtracting the rare-stimulus from the frequent-stimulus tracing. The exam was performed twice to verify reproducibility. These assessments followed the protocol described by Souza et al., 47 Didoné et al., 48 and McPherson. 27 The normality criteria were those proposed by McPherson. 27

Finally, three speech therapists with experience in evaluating auditory potentials and P3 auditory evoked potentials independently analyzed the results.

f) *Scale of Auditory Behaviors (SAB).* With the help of their guardians, children from both groups answered the Scale of Auditory Behaviors (SAB) questionnaire, translated into European Portuguese by Nunes, Pereira, and Carvalho, 49 to assess the child's communication deficits and the impact on their daily life.

### Statistical Analysis

A Shapiro-Wilk test was used to assess data normality. To verify the agreement of the analysis of the P2 and P3 waves, the Cohen Kappa coefficient was used. The correlation between the strength of agreement and the Kappa value was interpreted based on the ratings: < 0.00 (poor), 0.00 to 0.20 (negligible), 0.21 to 0.40 (weak), 0.41 to 0.60 (moderate), 0.61 to 0.80 (substantial), and 0.81 to 1.00 (almost perfect). The interpretation of the Interclass Correlation Coefficient (ICC) was based on the following classification: ICC < 0.4 (poor), ICC 0.4 to 0.75 (satisfactory), and ICC > 0.75 (excellent). Quantitative variables were described by means and standard deviations, and categorical variables by absolute and relative frequencies. A comparison of mean values between groups was performed using a student's *t* -test for independent groups in the case of symmetric variables, and a Mann-Whitney test for asymmetric variables. The results were computed using the IBM SPSS Statistics for Windows (IBM Corp., Armonk, NY, USA) software, version 23.0, and the figures were generated in R version 4.0.3, accessed via RStudio version 1.3.1093 (Posit PBC, Boston, MA, USA), using the ggplot2 package. A level of 5% was adopted as the criterion for statistical significance ( *p* ≤ 0.05).

## Result

### Sample Characterization

The current research involved 36 children (age group: 7–11 years; mean age: 8.89 years) divided into 2 groups, a CG and an EG, matched by sex, age, and handedness. Table 1 characterizes the sample data.

**Table 1:** Sample characterization

Parameter	Experimental group ( *n* = 18)	Control group ( *n* = 18)
Age: minimum–maximum (mean)	7–11 (8.89)	7–11 (8.89)
Sex: n (%)		
Male	16 (88.9%)	16 (88.9%)
Female	2 (11.1%)	2 (11.1%)
Manual preference: n(%)		
Right-handed	17 (94.4%)	17 (94.4%)
Left-handed	1 (5.6%)	1 (5.6%)
Stuttering severity: n(%)		
Mild	11 (61.1%)	0
Moderate	6 (33.3%)	0
Severe	1 (5.6%)	0

There was a predominance of males (88.9%) and right-handed children (94.4%). In terms of stuttering severity, 11 children (61.1%) in the EG had mild stuttering, 6 (33.3%) moderate stuttering, and 1 (5.6%) had stuttering classified as severe.

### Behavioral Assessment of Central Auditory Processing

Table 2 describes the results of behavioral assessments of central auditory processing skill. There was a significant difference between groups for the RGDT, DDT, DPT, and PSI tests.

**Table 2:** Behavioral assessment of auditory processing in children with and without stuttering

Behavioral tests	Experimental group ( *n* = 18): n (%)	Control group ( *n* = 18): n (%)	*p* -value
RGDT			
Normal	1 (5.6)	18 (100)	< 0.001*
Abnormal	17 (94.4)	0	
DDT			
Normal	9 (50)	18 (100)	0.001
Abnormal	9 (50)	0	
DPT			
Normal	4 (22.2)	18 (100)	< 0.001*
Abnormal	14 (77.8)	0	
PSI			
Normal	12 (66.7)	18 (100)	0.019*
Abnormal	6 (33.3)	0	
MLD			
Normal	17 (94.4)	18 (100)	1.000
Abnormal	1 (5.6)	0	

**Abbreviations** : DDT, dichotic digits test; DPT, duration pattern test; MLD, masking level difference; PSI, pediatric speech intelligibility; RGDT, random gap detection test.

Notes: Statistical significance (Fisher's exact test).

### Electrophysiological Evaluation

Table 3 presents the latency and amplitude results for waves P2 and P3. Statistically significant differences were observed regarding the latency of the P2 and P3 waves. For the P3 wave, statistically significant differences were observed in terms of both amplitude ( *p* < 0.001) and latency ( *p* < 0.001), in which there were longer latencies and smaller amplitudes in the group of stutterers.

**Table 3:** Values of P2 and P3 amplitude and latency in children with and without stuttering

Latency and amplitude	Groups				*p* -value ^§^
	Experimental group ( *n* = 18)		Control group ( *n* = 18)		
	Mean	SD	Mean	SD	
Latency P2 RE	175.3	15.7	164.6	14.2	0.040*
Latency P2 LE	176.0	15.5	164.4	14.2	0.026*
Latency P3 RE	373.6	52.8	310.2	41.6	< 0.001*
Latency P3 LE	375.6	46.7	309.5	42.4	< 0.001*
Amplitude P2 RE	6.4	1.4	6.2	1.1	0.706
Amplitude P2 LE	6.4	1.4	6.3	0.9	0.724
Amplitude P3 RE	17.8	2.8	23.3	2.8	< 0.001*
Amplitude P3 LE	17.8	3.1	24.4	4.2	< 0.001*

**Abbreviations** : LE, left ear; RE, right ear; SD, standard deviation.

Notes: §, Student's *t* -test; *statistically significant.

### Comparison between Behavioral and Electrophysiological Assessments

**Fig. 1:**
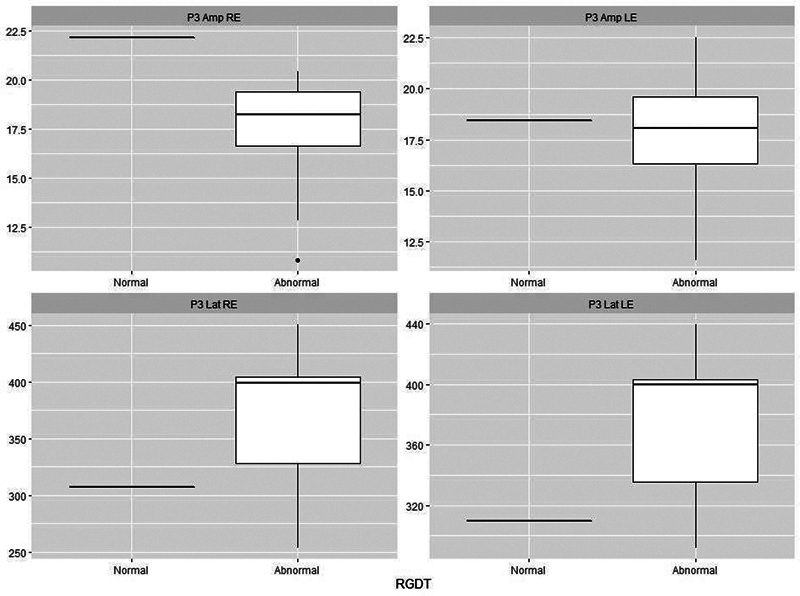
Plots of P3 parameters for 18 stutterers based on whether they were classified as normal ( *n* = 1) or abnormal ( *n* = 17) on the Random Gap Detection Test (RGDT). Abbreviations: Lat, latency (ms); Amp, amplitude (μV); RE, right ear; LE, left ear.

Figs. 1^2^,3,4,5 present the P3 electrophysiological findings for children who stutter, divided into those who passed a particular behavioral assessment and those who failed it (as set out in Table 2 ). On this basis, it is possible to see that there is a clear separation between the two categories for both the DTT and the DPT.

**Fig. 2:**
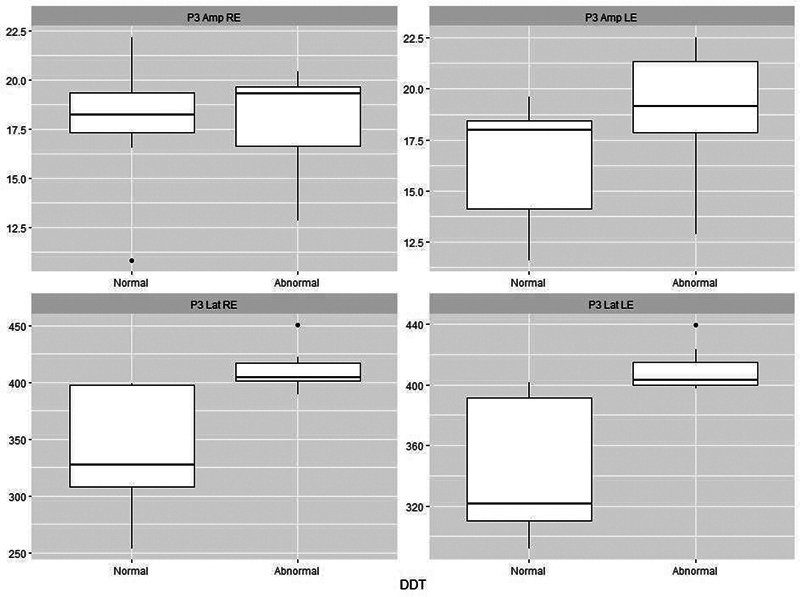
Plots of P3 parameters for 18 stutterers based on whether they were classified as normal ( *n* = 9) or abnormal ( *n* = 9) on the Dichotic Digit Test (DDT). There is a clear separation between the two classes in terms of latency. Abbreviations as per Fig. 1.

**Fig. 3:**
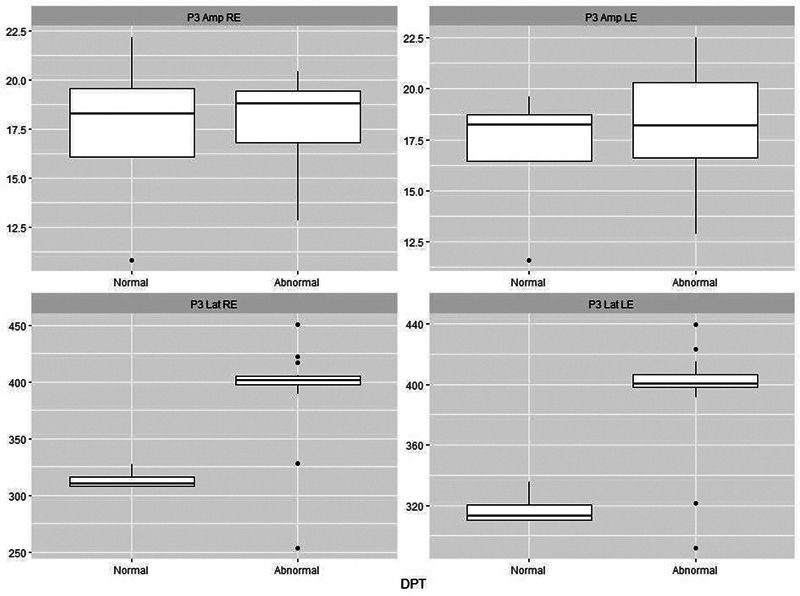
Plots of P3 parameters for 18 stutterers based on whether they were classified as normal ( *n* = 4) or abnormal ( *n* = 14) on the Duration Pattern Test (DPT). There is a clear separation between the two classes in terms of latency. Abbreviations as per Fig. 1.

**Fig. 4:**
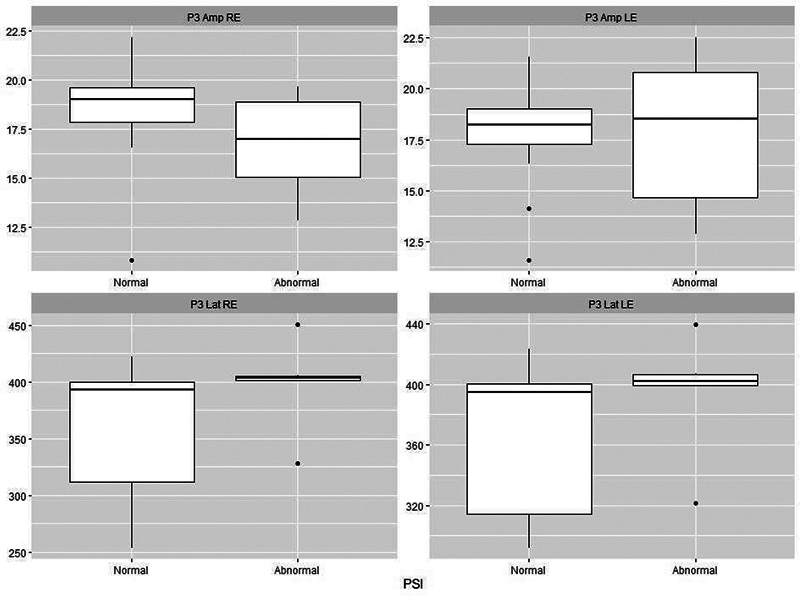
Plots of P3 parameters for 18 stutterers based on whether they were classified as normal ( *n* = 12) or abnormal ( *n* = 6) on the Pediatric Speech Intelligibility test (PSI). Abbreviations as per Fig. 1.

**Fig. 5:**
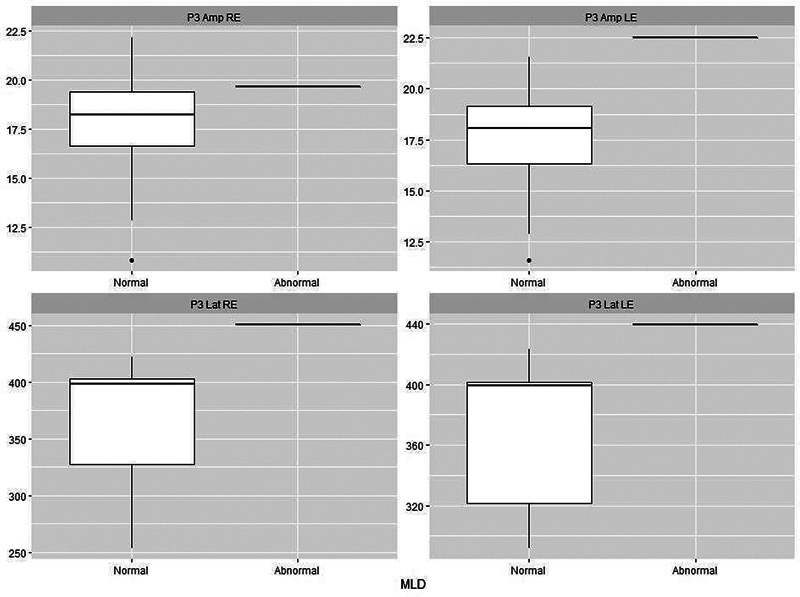
Plots of P3 parameters for stutterers based on whether they were classified as normal ( *n* = 17) or abnormal ( *n* = 1) on the Masking Level Difference test (MLD). Abbreviations as per Fig. 1.

### Agreement between Judges

There was excellent agreement between judges in the analysis of components of the P2 (Kappa = 0.88) and P3 (Kappa = 0.85) waves, as well as in the analysis of stuttering severity (Kappa = 0.82). Regarding the ICC, an ICC of 0.85 was obtained for P2 and 0.88 for P3, meaning almost perfect correlation.

### Stuttering Severity

Because only one patient presented severe stuttering, this data was not taken into consideration when looking for correlations between electrophysiological findings and stuttering severity. However, in terms of those children with mild or moderate stuttering, a positive association was found between stuttering severity and increased latency of waves P2 ( *p* < 0.001 in both ears) and P3 (RE: *p* = 0.015; LE: *p* = 0.048). This association was not found in the amplitude variable: for P2 the *p* -value for the RE was *p* = 0.216, and for the LE it was *p* = 0.180; similarly, for P3 the *p* -value for the RE was *p* = 0.098 and for the LE it was *p* = 0.301.

### Scale of Auditory Behaviors

In the analysis of scores on the SAB questionnaire, all the children in the CG fell into the typical component category. However, in the EG, 10 (55.6%) children were typical, but 7 (38.9%) had low risk, and 1 (5.6%) had high risk of auditory processing alterations. Since only 1 child had high risk of auditory processing alterations, the correlation analysis between SAB scores and electrophysiological assessments was performed only with children having either a typical component or a low risk for auditory processing alterations. An association was found between SAB score and latency of waves P2 and P3, but not for P2 and P3 amplitude. The numbers for P2 latency were: RE, *p* = 0.025; LE, *p* = 0.043. For P3 latency, the corresponding figures were: RE, *p* = 0.007; LE, *p* = 0.003. The non-significant amplitude figures were for P2 (RE, *p* = 0.270; LE, *p* = 0.364) and for P3 (RE, *p* = 0.536; LE, *p* = 0.669).

## Discussion

Stuttering, like other language disorders, largely affects males, 4 7 and this was observed in the present study, in which 88.9% of the stuttering group was made up of boys.

In our results we found that both P2 and P3 had longer latencies in the experimental group—the stutterers—compared with the control group ( Table 3 ). Similar findings have previously been found in adults 39 and in children, 40 indicating that stutterers may have a reduced cortical representation of acoustic stimuli and need more time to detect and discriminate sound inputs.

Regarding the amplitude of the P2 wave, no significant difference was observed between groups. One study performed on children who stutter found an increase in P2 amplitude only in the right ear 40 whereas another found a decrease in amplitude in adults. 39 No other studies appear in the reviewed literature which analyzed the amplitude of the P2 wave in children who stutter. In the sample studied, no change was observed in the amplitude of the P2 wave in children who stutter, indicating that children who stutter do not show a difference in the number of responsive neurons recruited to perform the task. It is suggested that further studies be performed on this topic.

Turning to P3, when results from the EG and CG were compared, differences were observed in terms of both the latency and amplitude of this wave, regardless of the ear stimulated ( Table 3 ). Children who stuttered showed longer latencies, corroborating what is found in the literature. 19 38 39 40 41 These findings indicate that stutterers perceive acoustic parameters with less precision and with longer reaction times due to some defect in acoustic processing. Similarly, as described in the literature, 19 38 39 40 41 children from the EG had lower P3 amplitudes compared with those in the CG ( Table 3 ). This may indicate that children who stutter require a greater neuron activation to differentiate and interpret auditory stimuli.

When non-linguistic stimuli are used in an oddball paradigm, P3 measurements reflect attention and memory skills. 24 35 40 Therefore, the findings of the present study suggest that the physiological processes devoted to attention and working memory are less robust in children who stutter. Previous studies of the performance of stutterers on behavioral tasks suggest that such individuals have difficulty focusing, regulating and sustaining attention, controlling attention in the presence of distracting background stimuli and conversations, and resisting or recovering from distractions. 50 51 52

Regarding the behavioral assessment of central auditory processing skills (the RGDT, DDT, DPT, and PSI), the present study found lower performance of the EG when compared with the CG. This demonstrates that stutterers have difficulty in temporal resolution, figure-ground discrimination, binaural separation, duration discrimination, temporal ordering, and recognition of verbal sounds in monotic and dichotic listening. Other studies have also found deficits in behavioral assessments of central auditory processing in individuals who stutter. 18 19 20 21 22 23 53 It is believed that this inability to process information can contribute to the difficulty that people who stutter have in maintaining fluent speech.

In the comparison between the electrophysiological tests and behavioral findings in the EG, it was possible to see that there was a clear demarcation between P3 and the DPT and DDT ( Figs. 2 3 ), demonstrating that an increase in P3 latency is a strong indication of difficulties in acquiring or storing sequential information. 54 Individuals who stutter seem to need more time to elicit the P3 component, which impacts the speed of auditory sound processing, and this may explain the changes found here in DPT.

Since duration is an acoustic parameter that underlies the processing of uttered speech and heard voice, it is intimately connected to the rhythm and rate of speech. 18 Thus, children who stutter seem to have difficulty extracting the supra-segmental aspects of speech, and this may be a factor in causing disfluent speech.

Turning to the severity of stuttering, the majority of our EG was made up of children with mild and moderate levels of stuttering. A positive association was found between stuttering severity and increased latency of waves P2 and P3, suggesting that attention, discrimination, and memorization skills of acoustic stimuli may be related to speech fluency. A previous study performed with behavioral tests showed that the severity of stuttering is directly related to performance in tests of non-verbal auditory processing. 21 However, another study 55 failed to find the same correlation. In a literature search, we found no other studies that correlated the severity of stuttering with electrophysiological assessments.

Finally, regarding the results obtained on the SAB, we found that most children in the EG were identified as having typical auditory responses or having a low risk of alterations in their auditory processing. When the SAB scores were compared with the electrophysiological assessments, a positive correlation was observed between the SAB score and the latency of waves P2 and P3, Nevertheless, there is a need for more research in this area before one can say such a correlation is present in children at high risk of changes in auditory processing.

## Conclusion

The findings of the present study provide evidence through electrophysiological and behavioral assessments that children who stutter may present changes in their abilities related to central auditory processing. The changes in latency and amplitude found in the analysis of P2 and P3 waves suggest that, even when not speaking, children who stutter exhibit electrophysiological differences in their AEPs. Knowledge about hearing difficulties in this population could contribute to the development of better therapeutic plans, including auditory training, with a view to improving auditory skills and improving speech fluency.

## References

[1] Wingate M E A standard definition of stuttering J Speech Hear Disord 1964 29 484 489 14257050 10.1044/jshd.2904.484

[2] Chang S E Zhu D C Neural network connectivity differences in children who stutter Brain 2013 136(Pt 12):3709 3726 24131593 10.1093/brain/awt275 PMC3859219

[3] Oliveira C M Bohnen A J Diagnóstico Diferencial dos Distúrbios de Fluência Ribeirão Preto Book Toy 2017 5 82

[4] Yairi E Ambrose N Epidemiology of stuttering: 21st century advances J Fluency Disord 2013 38 02 66 87 23773662 10.1016/j.jfludis.2012.11.002 PMC3687212

[5] Reilly S Onslow M Packman A Natural history of stuttering to 4 years of age: a prospective community-based study Pediatrics 2013 132 03 460 467 23979093 10.1542/peds.2012-3067

[6] Andrade C R Gagueira desenvolvimental persistente Barueri Pró-Fono 2017 5 10

[7] Degiovani V M Chiari B M Schiefer A M Disfluência: caracterização dos tipos e frequência de ocorrência em um grupo de escolares Pró-Fono R Atual Cient 1999 11 01 32 7

[8] Ingham R J Fox P T Ingham J C Functional-lesion investigation of developmental stuttering with positron emission tomography J Speech Hear Res 1996 39 06 1208 1227 8959606 10.1044/jshr.3906.1208

[9] Kroll R M De Nil L F Neural bases of stuttering and its treatment Memphis TN 2000

[10] Sandak R Fiez J A Stuttering: a view from neuroimaging Lancet 2000 356(9228):445 446 10981883 10.1016/S0140-6736(00)02547-2

[11] Van Borsel J Sunaert R Engelen S Speech disruption under delayed auditory feedback in multilingual speakers J Fluency Disord 2005 30 03 201 217 16038967 10.1016/j.jfludis.2005.05.001

[12] Brown S Ingham R J Ingham J C Laird A R Fox P T Stuttered and fluent speech production: an ALE meta-analysis of functional neuroimaging studies Hum Brain Mapp 2005 25 01 105 117 15846815 10.1002/hbm.20140 PMC6871755

[13] Chang S E Zhu D C Choo A L Angstadt M White matter neuroanatomical differences in young children who stutter Brain 2015 138(Pt 3):694 711 25619509 10.1093/brain/awu400 PMC4339778

[14] Weber-Fox C Hampton Wray A Arnold H Early childhood stuttering and electrophysiological indices of language processing J Fluency Disord 2013 38 02 206 221 23773672 10.1016/j.jfludis.2013.01.001 PMC3687214

[15] Desai J Huo Y Wang Z Reduced perfusion in Broca's area in developmental stuttering Hum Brain Mapp 2017 38 04 1865 1874 28035724 10.1002/hbm.23487 PMC5342907

[16] Pereira L D Navas A LGP Santos M TM Processamento auditivo: uma abordagem de associação entre a audição e a linguagem Barueri Manole 2002 75 95

[17] Costa J B Ritto A P Juste F S Andrade C RF Comparison between the speech performance of fluent speakers and individuals who stutter CoDAS 2017 29 02 e20160136 28327784 10.1590/2317-1782/20172016136

[18] Silva R Oliveira C MC Cardoso A CV Aplicação dos testes de padrão temporal em crianças com gagueira desenvolvimental persistente Rev CEFAC 2011 13 902 908

[19] Prestes R de Andrade A N Santos R B Marangoni A T Schiefer A M Gil D Temporal processing and long-latency auditory evoked potential in stutterers Braz J Otorhinolaryngol 2017 83 02 142 146 27233690 10.1016/j.bjorl.2016.02.015 PMC9442719

[20] Blood I M Disruptions in auditory and temporal processing in adults who stutter Percept Mot Skills 1996 82 01 272 274 8668488 10.2466/pms.1996.82.1.272

[21] Andrade C RF Schochat E Comparação entre os achados neurolinguísticos e neuroaudiológicos das gagueiras Pró-fono R Atual Cient 1999 11 27 30

[22] Andrade N A Gil D Schiefer A M Pereira L D Processamento auditivo em gagos: análise do desempenho das orelhas direita e esquerda Rev Soc Bras Fonoaudiol 2008 13 01 20 29

[23] Andrade A N Gil D Schiefer A M Pereira L D Avaliação comportamental do processamento auditivo em indivíduos gagos Pró-Fono R Atual Cient 2008 20 01 43 8. (2008b) 10.1590/s0104-56872008000100008 18408863

[24] Hall J W P300 response Florida Allyn & Bacon 2006 518 47

[25] Kamita M K Silva L AF Matas C G Potenciais evocados auditivos corticais no transtorno do espectro do autismo: revisão sistemática CoDAS 2021 33 02 e20190207 34037100 10.1590/2317-1782/20202019207

[26] Frizzo A CFA Potenciais evocados auditivos de longa latência: conceitos e aplicações clínicas São Paulo Book Toy 2018 139 50

[27] McPherson D L Late potentials of the auditory system San Diego Singular Publishing Group 1996

[28] Martin B A Tremblay K L Stapells D R Principles and applications of cortical auditory evoked potentials Baltimore Lippincott Williams and Wilkins 2007 482 507

[29] Almeqbel A Speech-evoked cortical auditory responses in children with normal hearing S Afr J Commun Disord 2013 60 38 43 25158372

[30] Tremblay K L Ross B Inoue K McClannahan K Collet G Is the auditory evoked P2 response a biomarker of learning? Front Syst Neurosci 2014 8 28 24600358 10.3389/fnsys.2014.00028 PMC3929834

[31] García-Larrea L Lukaszewicz A C Mauguière F Revisiting the oddball paradigm. Non-target vs neutral stimuli and the evaluation of ERP attentional effects Neuropsychologia 1992 30 08 723 741 1407488 10.1016/0028-3932(92)90042-k

[32] Musiek F E Froke R Weihing J The auditory P300 at or near threshold J Am Acad Audiol 2005 16 09 698 707 16515141 10.3766/jaaa.16.9.7

[33] Sleifer P Avaliação eletrofisiológica da audição em crianças Rio de Janeiro Revinter 2015 171 94

[34] Sanfins M D Matas C G Potencial Evocado Auditivo de Longa Latência (Peall): Potencial Cognitivo (P300) Ribeirão Preto, São Paulo Book Toy 2022 259

[35] Jaeger A Parente M AMP Cognição e eletrofisiologia: uma revisão crítica das perspectivas nacionais Psico-USF 2010 15 02 171 180

[36] Angrisani R MG Matas C G Neves I F Sassi F C Andrade C RF Avaliação eletrofisiológica da audição em gagos, pré e pós terapia fonoaudiológica Pró-Fono R Atual Cient 2009 21 95 100 10.1590/s0104-56872009000200002 19629317

[37] Khedr E El-Nasser W A Abdel Haleem E K Bakr M S Trakhan M N Evoked potentials and electroencephalography in stuttering Folia Phoniatr Logop 2000 52 04 178 186 10782010 10.1159/000021532

[38] Sassi F C Matas C G de Mendonça L I de Andrade C R Stuttering treatment control using P300 event-related potentials J Fluency Disord 2011 36 02 130 138 21664531 10.1016/j.jfludis.2011.04.006

[39] Hampton A Weber-Fox C Non-linguistic auditory processing in stuttering: evidence from behavior and event-related brain potentials J Fluency Disord 2008 33 04 253 273 19328979 10.1016/j.jfludis.2008.08.001 PMC2663969

[40] Regaçone S F Stenico M B Gução A CB Rocha A CM Avaliação eletrofisiológica do sistema auditivo em indivíduos com gagueira desenvolvimental persistente Rev CEFAC 2015 17 06 1838 1847

[41] Jerônimo G M Scherer A PR Sleifer P Potenciais evocados auditivos de longa latência em crianças com gagueira Einstein (Sao Paulo) 2020 18 1 6

[42] Maxfield N D Olsen W L Kleinman D Frisch S A Ferreira V S Lister J J Attention demands of language production in adults who stutter Clin Neurophysiol 2016 127 04 1942 1960 26971476 10.1016/j.clinph.2016.01.016 PMC4792332

[43] Andrade C RF Befi-Lopes D M Fernandes F DM Wertzner H F ABFW: teste de linguagem infantil nas áreas de fonologia, vocabulário, fluência e pragmática Barueri Pró Fono 2004

[44] Andrade C RF Protocolo para avaliação da fluência da fala Pró-Fono R Atual Cient 2000 12 02 131 4

[45] Riley G Stuttering Severity Instrument (SSI-4) Austin Pro-Ed 2009

[46] ABA Academia Brasileira de Audiologia. Fórum: diagnóstico audiológico – 2016 São Paulo ABA 2016

[47] Souza J Rocha V O Berticelli A Z Didoné D D Sleifer P Auditory latency response - P3 in children with and without learning complaints Audiol Commun Res 2017 22 e1690

[48] Didoné D D Garcia M V Oppitz S J Potencial evocado auditivo P300 em adultos: valores de referência Einstein (Sao Paulo) 2016 14 02 208 212 27462895 10.1590/S1679-45082016AO3586 PMC4943355

[49] Nunes C L Pereira L D Carvalho G S Scale of Auditory Behaviors e testes auditivos comportamentais para avaliação do processamento auditivo em crianças falantes do português europeu CoDAS 2013 25 03 209 215 24408330 10.1590/s2317-17822013000300004

[50] Anderson J D Wagovich S A Aspects of attention in developmental stuttering. Presented at the American Speech-Language-Hearing Association (ASHA) Philadelphia 2016

[51] Eichorn N Marton K Pirutinsky S Cognitive flexibility in preschool children with and without stuttering disorders J Fluency Disord 2018 57 37 50 29157666 10.1016/j.jfludis.2017.11.001

[52] Ofoe L C Anderson J D Ntourou K Short-Term Memory, Inhibition, and Attention in Developmental Stuttering: A Meta-Analysis J Speech Lang Hear Res 2018 61 07 1626 1648 29984373 10.1044/2018_JSLHR-S-17-0372 PMC6195058

[53] Beinisis E Resolução temporal em indivíduos com e sem gagueira São Paulo Universidade Federal de São Paulo 2012

[54] Pereira L D Sistema auditivo e desenvolvimento das habilidades auditivas São Paulo Roca 2004 547 52

[55] Schiefer A M Pereira L D Barbosa L G Considerações preliminares entre uma possível correlação entre gagueira e os aspectos linguísticos e auditivos Pró-Fono R Atual Cient 1999 11 01 27 31

